# Rationalized Development of a Campus-Wide Cell Line Dataset for Implementation in the Biobank LIMS System at Bioresource Center Ghent

**DOI:** 10.3389/fmed.2019.00137

**Published:** 2019-06-25

**Authors:** Veronique T'Joen, Lieven Vaneeckhaute, Sara Priem, Steven Van Woensel, Sofie Bekaert, Elke Berneel, Catherine Van Der Straeten

**Affiliations:** ^1^Bioresource Center Ghent, Health, Innovation and Research Center, Ghent University Hospital, Ghent, Belgium; ^2^Data Management Unit, Health, Innovation and Research Center, Ghent University Hospital, Ghent, Belgium; ^3^Department of Public Health and Primary Care, Faculty for Medicine and Health Sciences, Ghent University, Ghent, Belgium; ^4^Health, Innovation and Research Center, Ghent University Hospital, Ghent, Belgium

**Keywords:** biobank, cell line, data management system, dataset, quality, data quality

## Abstract

The Bioresource center Ghent is the central hospital-integrated biobank of Ghent University Hospital. Our mission is to facilitate translational biomedical research by collecting, storing and providing high quality biospecimens to researchers. Several of our biobank partners store large amounts of cell lines. As cell lines are highly important both in basic research and preclinical screening phases, good annotation, authentication, and quality of these cell lines is pivotal in translational biomedical science. A Biobank Information Management System (BIMS) was implemented as sample and data management system for human bodily material. The samples are annotated by the use of defined datasets, based on the BRISQ (Biospecimen Reporting for Improved Study Quality) and Minimum Information About Biobank data Sharing (MIABIS) guidelines completed with SPREC (Standard PREanalytical Coding) information. However, the defined dataset for human bodily material is not ideal to capture the specific cell line data. Therefore, we set out to develop a rationalized cell line dataset. Through comparison of different datasets of online cell banks (human, animal, and stem cell), we established an extended cell line dataset of 156 data fields that was further analyzed until a smaller dataset—the survey dataset of 54 data fields—was obtained. The survey dataset was spread throughout our campus to all cell line users to rationalize the fields of the dataset and their potential use. Analysis of the survey data revealed only small differences in preferences in data fields between human, animal, and stem cell lines. Hence, one essential dataset for human, animal and stem cell lines was compiled consisting of 33 data fields. The essential dataset was prepared for implementation in our BIMS system. Good Clinical Data Management Practices formed the basis of our decisions in the implementation phase. Known standards, reference lists and ontologies (such as ICD-10-CM, animal taxonomy, cell line ontology…) were considered. The semantics of the data fields were clearly defined, enhancing the data quality of the stored cell lines. Therefore, we created an essential cell line dataset with defined data fields, useable for multiple cell line users.

## Introduction

In the last two decades, biobanks—specialized infrastructures that store, annotate, and distribute biospecimens—have emerged and professionalized through the implementation of quality and data management systems based on harmonized minimal datasets which allow sharing of samples between researchers and thus enhancing progression of clinical research (1).

In 2015, the Bioresource center Ghent—formerly known as Bimetra Biobank (2)—established a central high quality biobanking facility at Ghent University hospital. This hospital-integrated biobank brought together multiple decentralized biobank initiatives into a professionalized biobank, with implemented quality management system.

Local strategic prospective collections, important historical collections, and interuniversity focus collections are operationally managed within the biobank through an implemented biobank information management system (BIMS), named SLims^1^. Current minimal datasets for these collections reflect recommended fields from known guidelines (3–6) or standards (7, 8) complemented with quality parameters, by use of the “Standard Pre-analytical Code” (SPREC) (9, 10) or the “Biospecimen Reporting for Improved Study Quality” (BRISQ) system (11, 12), as harmonization of datasets is still ongoing at the European (“Biobanking and Biomolecular Resources Research Infrastructure—European Research Infrastructure Consortium” (BBMRI-ERIC)^2^) and international (driven by the International Society for Biological and Environmental Repositories (ISBER)^3^) level. The samples are collected in a project-based manner and can be used for fundamental basic research studies, in preclinical screening phases and in actual clinical trials.

The Bioresource center Ghent is part of the “Health, innovation and research institute” of Ghent University Hospital, which is a central contact point, service provider and knowledge center for biomedical translational and clinical research and health care innovation. The goal of translational biomedical science—an interdisciplinary field—is to expedite health care progress in prevention, diagnosis and treatment by combining disciplines, resources, expertise and techniques (13). The mission of the Bioresource center Ghent is to operate as a central contact point, knowledge center and high-quality service provider for all aspects related to biobanking.

Translational biomedical science is a clinical domain supported by three main pillars: bench side, bedside and the community. The translation of “bench side” observations into actual clinical applications is a long and elaborate process. Before actual clinical trials can be initiated, several basic research and preclinical research phases have to be completed. In preclinical screening phases, potential chemical compounds are often screened in the lab on cell lines. Both human and animal derived cell lines are considered as representative model systems for studying numerous biological mechanisms and serve as important preclinical models for drug target discovery and rapid assessment of toxicity profiles (14).

Cell line annotation, authentication as well as the quality of the cell line are pivotal for determining the reliability and reproducibility of these preclinical tests. The lack of attention given to these preclinical data is an underestimated problem in biomedical science, leading to delays and increased costs in drug discovery studies (15, 16). Vast warehouses of cell line samples are available in commercial and academic settings. However, the datasets pertaining to these cell line samples differ massively in content and information (e.g., cell line origin, processing history) leading to cell line misidentification, misuse, mismatching, and the use of mixed clones by culture mix-ups (17, 18). Remarkably, SPREC and BRISQ do not cover specific data fields for cell lines, as they are categorized as complex derivatives, whose isolation requires usage of multiple steps and/or addition of chemical substances (3).

As multiple cell line collections are present on our campus, we set out to develop a uniform, campus-wide essential cell line dataset that tackles the issues regarding misidentification, annotation and poor culture follow-up. Our experience with cell lines indicated that a comprehensive cell line dataset should ideally contain three large categories of information.

First of all, general information regarding the origin and culture of the cell line, such as cell line name, type of tissue, derivation method, relevant clinical, and demographic information, cell line passage, current culture/freezing/thawing protocols and cell line aging information is paramount (19, 20).

Secondly, information for clear authentication of the cell line should be included. Cell line authentication relies on comparing samples derived from the same donor (16) by Short Tandem Repeat (STR) profiling and Single Nucleotide Polymorphisms analysis. To our current knowledge, there is no general approved standard or centralized online reference database for cell line authentication using Single Nucleotide Polymorphisms analysis (18, 21), leading to inaccuracy (22), although there is a general consensus on the need to establish this for cell line authentication.

Thirdly, quality data should be available, such as information regarding control of bacterial, viral, fungal of mycoplasma contaminations of the cell cultures (23, 24).

Thus, we set out to develop a comprehensive cell line dataset which would enhance cell line quality and their usability in translational research.

## Materials and Methods

### Establishment of the “Extensive Cell Line Parameter Dataset”

Relevant articles regarding cell line datasets were searched in PubMed®. Additionally, several cell line companies, vendors and a large cell line locator^4^ were identified through a general website search. A selection of frequently used and mentioned cell banks was made, taking into account that human (15 cell banks), animal (15 cell banks), and stem cell lines (3 cell banks) were represented within the selected banks. All data fields found in the cell banks were listed, forming the “Extensive cell line data field set.”

### Evaluation of the “Extensive Cell Line Data Field Set” and Establishment of the “Survey Cell Line Data Field Set”

The usability of the “Extensive Cell line data field set” for different cell types was evaluated by subdividing the dataset fields into 6 nominative categories (named “basic cell line,” “administrative information,” “clinical and demographic,” “cell culture,” “genetic” and “quality/validation data” fields) and comparing the presence of each data field per cell line type, thus for human, animal, and stem cell lines. Data fields that were hardly present in any cell line database were eliminated from the survey. Subsequently, a redundancy strategy was applied to the dataset in order to eliminate data fields in which similar and overlapping information was captured. To identify similar and overlapping information, the selected 15 human cell banks were searched for a particular widely used human kidney (HEK 293, immortalized human embryonic kidney cell line) and cancer (HeLa, immortalized cell line from cervical cancer cells) cell line. The 15 selected animal cell banks were searched for a particular well-known animal cell line [MC 3T3, osteoblast precursor cell line derived from Mus musculus (mouse) calvaria cell line]. The obtained information in each data field was listed and compared. Subsequently, the most appropriate name for the data field was selected to provide an as clear as possible content for the field.

### REDCap (Research Electronic Data Capture) Survey

The “Survey Parameter Set” formed the basis of a REDCap survey (25). Survey data were collected and managed using REDCap electronic data capture tools hosted at Ghent University Hospital^5^. REDCap (Research Electronic Data Capture) is a secure, web-based application designed to support data capture for research studies, providing: (1) an intuitive interface for validated data entry; (2) audit trails for tracking data manipulation and export procedures; (3) automated export procedures for seamless data downloads to common statistical packages; and 4) procedures for importing data from external sources. The survey was distributed “campus-wide” to research groups that have cell lines to their disposition. The survey was constructed in such a way that for each type of cell line (i.e., human, animal, cancer, and stem cell lines), researchers could indicate responses, thus allowing the identification of essential differences in the data fields per type of cell line. The researchers were asked which parameters they retain for their cell lines at present and if they annotate their cell lines. Additionally, they were asked which data fields they would find relevant to be mentioned in the campus-wide minimal dataset.

#### REDCap Survey Analysis

The returned survey data were thoroughly evaluated per cell line type (human/animal/stem cell). The data fields were as described before, regrouped in nominative categories to allow efficient analysis of the data: Basic cell line data, clinical and demographic data fields, cell culture data, genetic data, quality, and validation data and administrative data. Data fields were considered as highly relevant if more than 50% of the responders indicated it. Fields were considered as not relevant if more than 50% of responders indicated it. Global analysis of the REDCap survey results led to the inclusion of data fields in the rationalized “essential cell line dataset.”

### Essential Dataset: Defining the Cell Line Dataset Template

The data fields obtained in the “essential cell line dataset” were further evaluated and defined to allow the actual development of a cell line dataset template. Several steps were initiated and each data field was individually reviewed. Some fields from the essential dataset were split into multiple fields so that one type of data would be recorded per data field and not a combination of data, which is a general “Good Clinical Data Management Practice (GCDMP)” rule. This was also applied for registering units accompanying their specific values.

The “label” or “field name” of the data fields were screened for synonymy for which a correction was made by selecting the least ambiguous term as “survivor.” If, after selecting the least ambiguous term, the need for a better label still persisted, a new label was proposed and presented to a panel consisting of biobank data managers and two quality managers, which have extensive experience with cell lines and cell culture. The newly proposed terminology was compared to literature to ensure its validity. Next, the essential data fields were defined by using the best existing description and thus introducing definitions to the data fields. Definitions were chosen from SPREC (10), BRISQ (12), MIABIS (6), PubMed (MeSH) or by adjusting existing definitions (26, 27) to best suit a biobanking/clinical context in concurrence with propositions from the Good Clinical Practices and the General Data Protection Regulation^6^. If no suitable existing definition could be found, a new definition would be postulated. Because of the great value of consistency in semantics, existing definitions were always favored above newly established definitions and if necessary, existing terms were divided into better definable sub labels.

A full list of the withheld data fields and their definitions can be found in **Table 3**.

### Implementation of the Essential Cell Line Dataset Through Use of Ontologies and Lists Within the BIMS

Following the creation of essential data field labels and providing a definition for the desired content of the field, the actual field options for filing in the fields were reviewed in order to obtain clear and consistent data. To implement and secure good data practices, optimal use was made of “fixed choice” data fields, with the addition of options as “not performed,” “unknown” or “missing data.”

Several known standards and ontologies were evaluated for implementation: the “Cell Line Ontology” (28), “International Classification of Diseases for Oncology (29)” and the “Biological Classification (Taxonomy) for Class, Order and Species.” For date and time stamps, the ISO 8601 standard^7^ was implemented and all units were collected through the use of the “International System of Units” (30). Preference was given to known standards if these were practical in use. If no appropriate standard could be found or was deemed suitable for our setting, data fields with a well-defined fixed choice list were implemented. By the combination of standards and ontologies, all elements of essential sample information were collected and stored in a structured and well-approved manner.

## Results

### Establishment of the Different Datasets

The Extensive Cell line parameter dataset was established as described in the Materials and methods section. Datasets used by different companies and described in articles were extracted and listed for comparison. This led to a dataset of 156 different data fields, visible in Table 1. All data fields in Table 1 are listed alphabetically. Subsequently, we divided the data fields in nominative categories (“basic cell line,” “administrative information,” “clinical and demographic,” “cell culture,” “genetic”, and “quality/validation data”) and a redundancy strategy was applied to reduce overlapping data. This led to a reduction of 65% of fields, resulting in 54 remaining data fields. The most appropriate name was chosen for overlapping data fields and the resulting set formed the survey cell line parameter dataset (Table 2).

**Table 1:** Extensive cell line data field set.

**General cell line information**	**Administrative information**	**Clinical and demographic information**	**Culture method information**	**Validation and quality control information**	**Genetic information**
Achor-dependancy	Analyse certificate	Age	Acclimatation of cells	Bacteria	Antigen expression
Advantages	Applications + advice	Age at collection	Antibiotic resistance	Biosafety guidelines	Antigen expression (surface)
Alias	Available product formats	Case history	Antibiotics	Biosafety level	Cell line stability
Animal	Catalog number	Clinical data	Anticoagulant	DAPI	Cytogenetics
Brief description	Cell culture images	Diagnosis information	Atmosphere	Flow cytometry	Details karyotype
Cell line alias	Comments	Disease	Cell density (cells/cm^2^)	Fungi	DNA Fingerprint
Cell line biological properties	Compliance with regulations	Donation frequency	Cellular products	Hazard	ELISA
Cell line description	Compliance with standards	Donor criteria	CO2 concentration	Health hazards of liquid nitrogen	Genes expressed
Cell line origin	Delivery forms	Ethnicity	Complete growth medium	Microbiological culture	Genetic alteration
Cell type	Distribution	Ethnicity information	Cryovial	MSDS file	Genetics
Clonality	Effects	Gender	Culture conditions	Mycoplasma	Immunology
Genus	Images	Harvest of cells	Derivation	Personal protective equipment	Isoenzymes
Identity	Limited use	Histopathology	Doubling time	Safety precautions	Karyotype
Lifespan	Limited warranty	Metastasis	Freeze concentration	Sterility	Mutational status
Morphological character	MTA agreement	Organ of metastasis	Freeze medium	Sterility tests	Oncogene
Morphology	Name of depositor	Pathology	Incubation	Storage precautions	Pathway activation
Organism	Originator	Preparation organ	Medium	Tryptan-Blue exclusion	PCR assay
Species	Ownership + patents	Race	Medium renewal frequency	Validation assay	Profile
Species validation	Permissions And Restrictions	Screened before donation	Passage	Viable cell count	Receptor expression
Strain	Price	Sex	Passage number	Viruses	Receptors
Tissue	Provider	Tissue form	Protocol for cell culture		Reprogramming method
Tissue origin	References	Weight	Protocol for cell thaw		Reverse transcriptase
	Register		Protocol for culture medium preparation		RNA hybridization
	Regulation		Protocol for freezing cells		STR profile
	Related products		Protocol for maintenance		Transformation
	Shipped in		Protocol for subculturing		Tumorigenic
	Shipping table + distribution notes		Quantity and concentration		
	Video + resources		Required materials		
	Year of origin		Split ratio		
			Storage conditions		
			Storage temperature		
			Subcultivation ratio		
			Subculture routine		
			Subculturing		
			Subculturing protocol		
			Temperature		
			Thawing method		

**Table 2:** Survey cell line data field set.

**Data field**	**Nominative category**	**Data field**	**Nominative category**
Adhesion	Basic cell line	Growth medium Additives	Cell culture
Age	Clinical and demographic	Growth medium Composition	Cell culture
Amount and cell conc	Basic cell line	Illness	Clinical and demographic
Antibiotic resistance	Cell culture	Immunology	Genetic
Antibiotics	Cell culture	Isoenzyme validation	Quality/validation data
Anticoagulant use	Cell culture	Lot number registration	Cell culture
Antigen expression	Genetic	Medium renewal (sub cultivation)	Cell culture
Biosafety	Basic cell line	Microbial screening status	Quality/Validation data
Cell line stability	Cell culture	Morphology	Basic cell line
Cell type	Basic cell line	MTA agreement	Administrative information
Cellular products	Genetic	Mutational Status	Genetic
Clinical data	Clinical and demographic	Mycoplasma Screening	Quality/validation data
Conformity with regulations	Administrative information	Organism	Basic cell line
Cryovial type	Cell culture	Passage number	Basic cell line
Culture atmosphere	Cell culture	Patents and properties	Administrative information
Culture temperature	Cell culture	Receptor expression	Genetic
Cytogenetics	Genetic	Reprogramming method	Genetic
Derivation	Basic cell line	Short tandem repeat profile	Quality/validation data
Details karyotype	Genetic	Sub cultivation ratio + cell density	Cell culture
Dna fingerprint	Quality/validation data	Subculture protocol	Cell culture
Doubling time	Cell culture	Supplier registration	Cell culture
Ethnicity	Clinical and demographic	Thawing method	Cell culture
Freezing medium composition	Cell culture	Tissue origin	Basic cell line
Freezing storage temperature	Cell culture	Transformation	Basic cell line
Gender	Clinical and demographic	Tumor details	Genetic
Gene expression	Genetic	Tumor formation	Genetic
GMO status	Genetic	Viral quality control	Quality/validation data

### Survey Results

**Figure 1:**
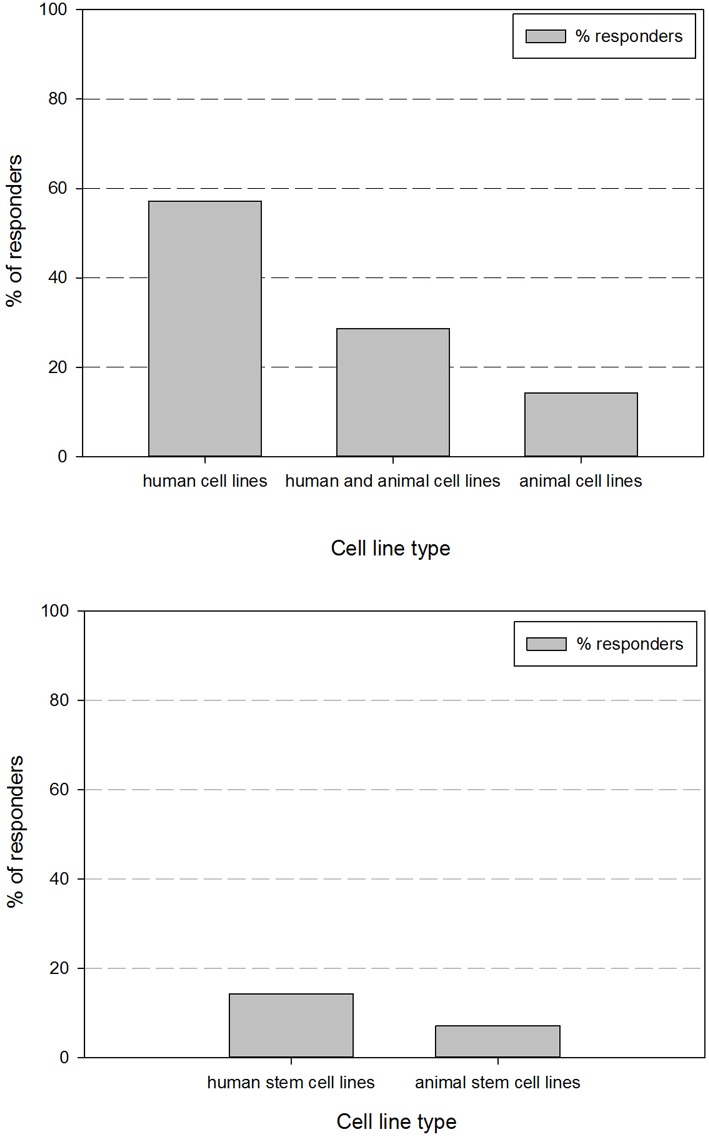
Overview of the percentage of responders vs. the type of cell line they manage.

A REDCap survey was designed using the survey cell line parameter dataset. The survey results cover the global responses of 17 different research groups on our campus. Figure 1 gives an overview of the received responders according to cell type origin. Responses showed that 57.1% of the respondents exclusively store human cell lines and 14.3% exclusively work with animal cell lines. 28.6% of the respondents work with both human and animal cell lines. Within the respondents that work with human cell lines (85.7%), all respondents have human cancer cell lines and 14.3% work additionally with human stem cell or induced pluripotent stem cell lines. Within the respondents that work with animal cell lines (42.9%), 7.1% work with animal stem cell or induced pluripotent stem cell lines.

**Figure 2:**
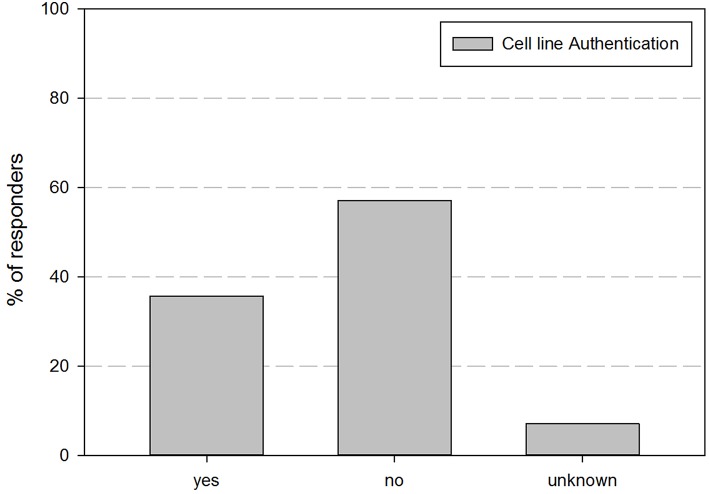
Overview of the percentage of responders that indicated to perform cell line authentication on their cell lines.

As cell line authentication is essential for good cell line practices, we also inquired if cell line authentication was performed before use of cell lines in experiments. Figure 2 shows their perspective regarding their performance of cell line authentication practices. This demonstrates that <35% of the responders authenticates the cell lines they are using.

Next, the survey data field list was analyzed per type of cell line, i.e., human, animal, and stem cell line. In order to be able to compare the results, the data fields were subdivided in 6 grouped categories: “basic cell line information,” “clinical and demographic data,” “cell culture information,” “genetic characteristics, quality,” “validation and administrative information.” The responders had the option to indicate if they found a field “Highly relevant,” “neutral” or “not relevant.” A cut-off point was set at 50%, meaning that if more than 50% of the responders found a field “highly relevant,” it should be included into the final dataset. Furthermore, if more than 50% of the responders found the field “not relevant,” it will not be include in the final dataset.

**Figure 3:**
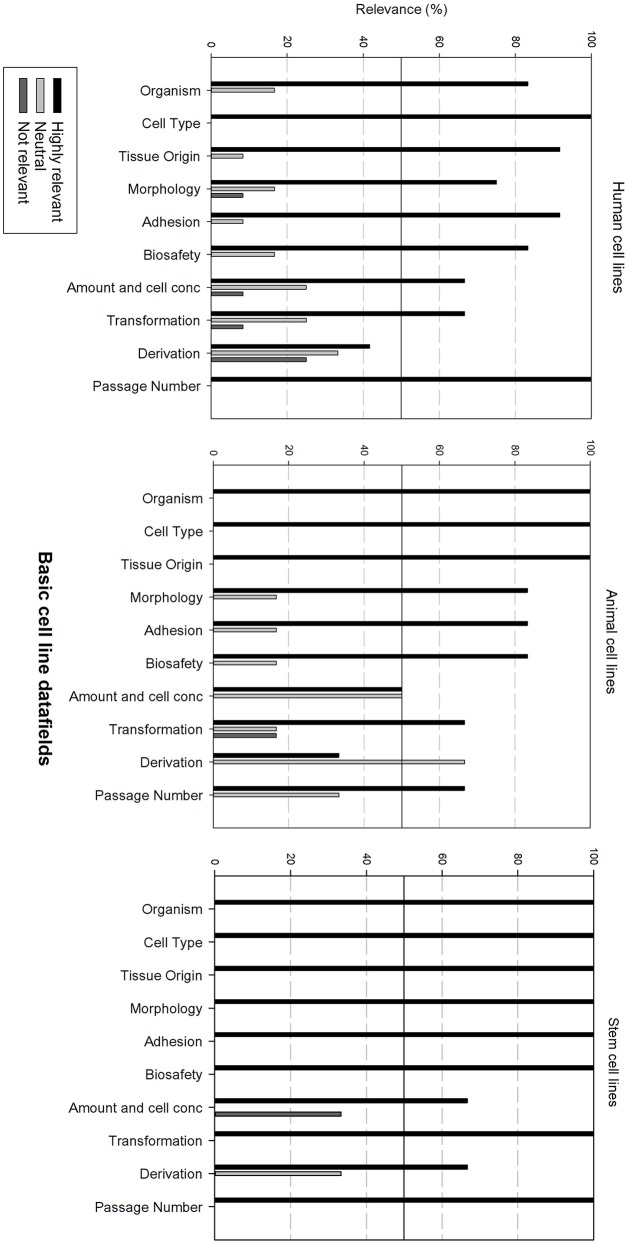
Overview of the relevance score of basic cell line data fields for each type of cell line. A cut-off line at 50% is visible, indicating which data fields are generally considered as highly relevant or as not relevant.

Figure 3 gives an overview of the relevance scores for the basic cell line data fields. A line was used for indicating the 50% relevance cut-off point. Analysis of the basic cell line data fields shows that most fields are considered as highly relevant, regardless of the cell line type. As can be seen, the fields “Cell type,” “Organism” and “Tissue origin” got the overall best relevance score. “Cell type” was the only field with a perfect score over the 3 types of cell lines. The field “derivation” is considered as neutral for human and animal cell lines, though highly relevant for stem cell lines. “Amount and cell conc” divides researchers of animal cell lines between “Neutral” and “Highly relevant.” Overall could be noted that these fields are extremely relevant for stem cell lines. Eight of the ten fields received a 100% highly relevant score, however all fields received a good to excellent score for all three types of cell lines.

**Figure 4:**
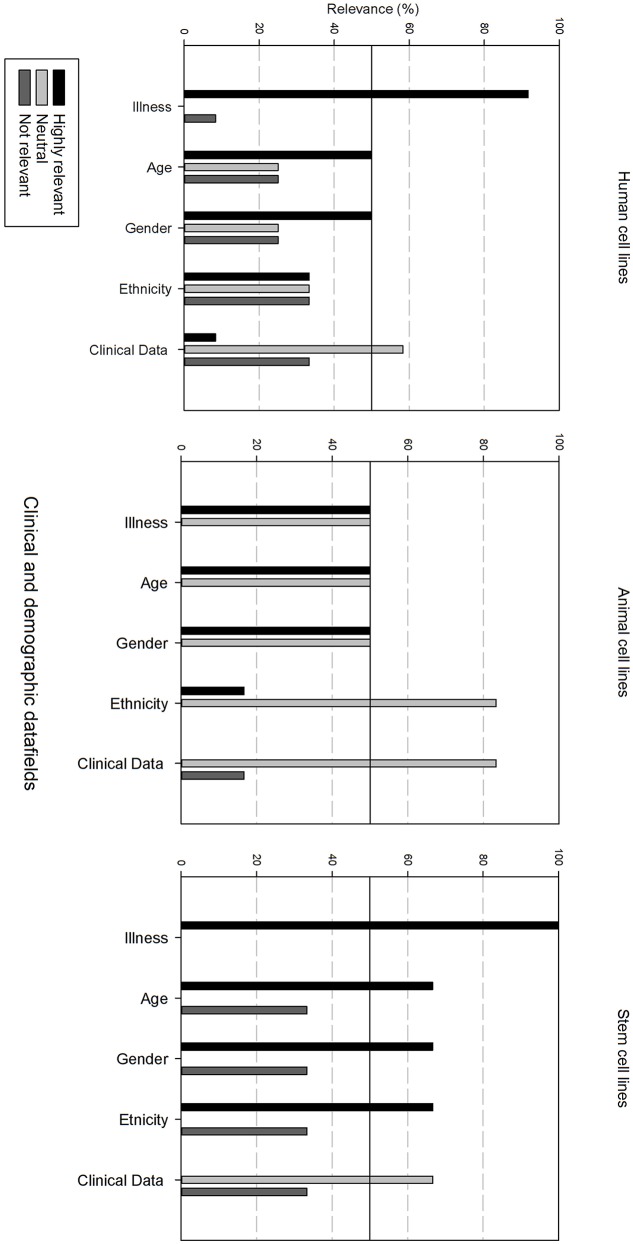
Overview of the relevance score of clinical and demographic data fields for each type of cell line. A cut-off line at 50% is visible, indicating which data fields are generally considered as highly relevant or as not relevant.

Figure 4 gives an overview of the clinical and demographic data fields. Within the clinical and demographic data, regardless of cell line type, “Illness, Age and Gender” are considered as highly relevant fields. Differences in relevance of the datafields can be seen depending on the type of cell line. Ethnicity is only viewed as highly relevant for stem cell lines, and as neutral for human and animal cell lines. Additional clinical and demographic data fields are seen as neutral.

**Figure 5:**
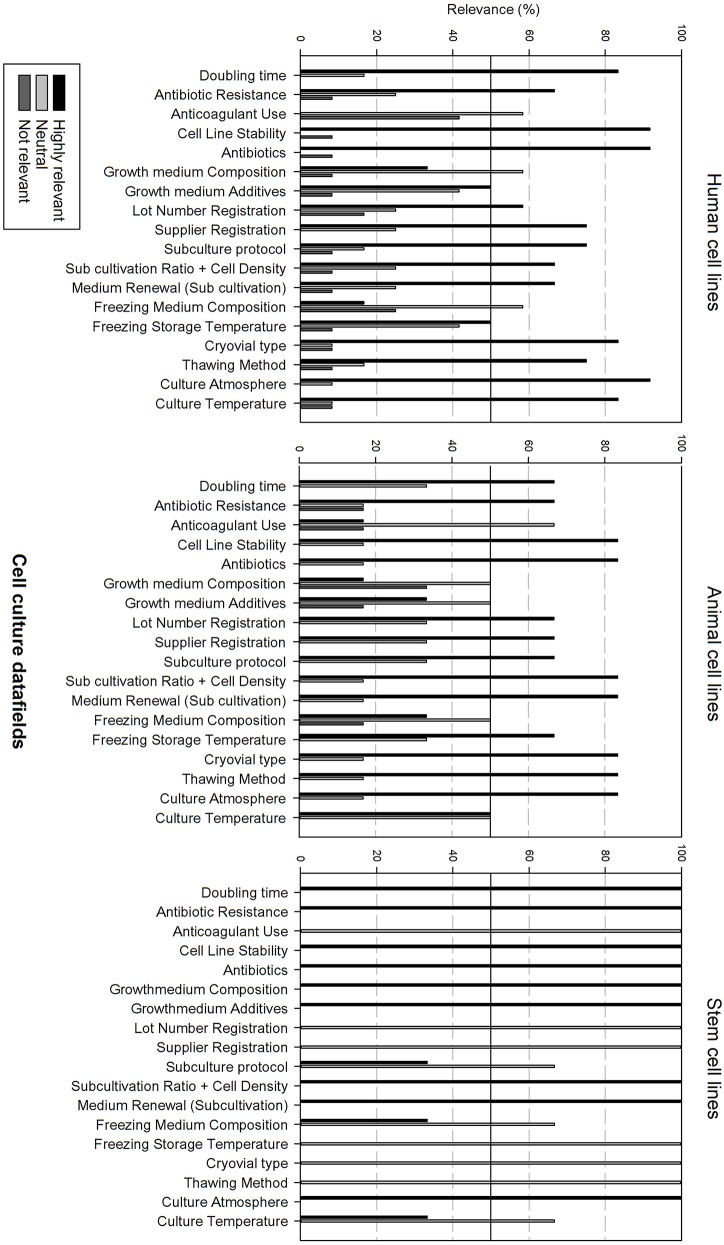
Overview of the relevance score of cell culture data fields for each type of cell line. A cut-off line at 50% is visible, indicating which data fields are generally considered as highly relevant or as not relevant.

Figure 5 gives an overview of the cell culture datafields. Differences can be observed between the cell line types. Most data fields (15 out of 18) are considered as highly relevant for human cell lines, except for anticoagulant use, growth medium, and freezing medium composition that are considered as neutral. Data analysis for animal cell lines is almost identical with the sole exception that growth medium additives are also considered as neutral. The relevance of cell culture data fields for stem cell lines differs, showing that only 9 out of 18 data fields are considered as highly relevant. Neutral data fields are: anticoagulant use, lot number registration, supplier registration, subculture protocol, freezing medium composition, freezing storage temperature, cryovial type, thawing method, and culture temperature.

**Figure 6:**
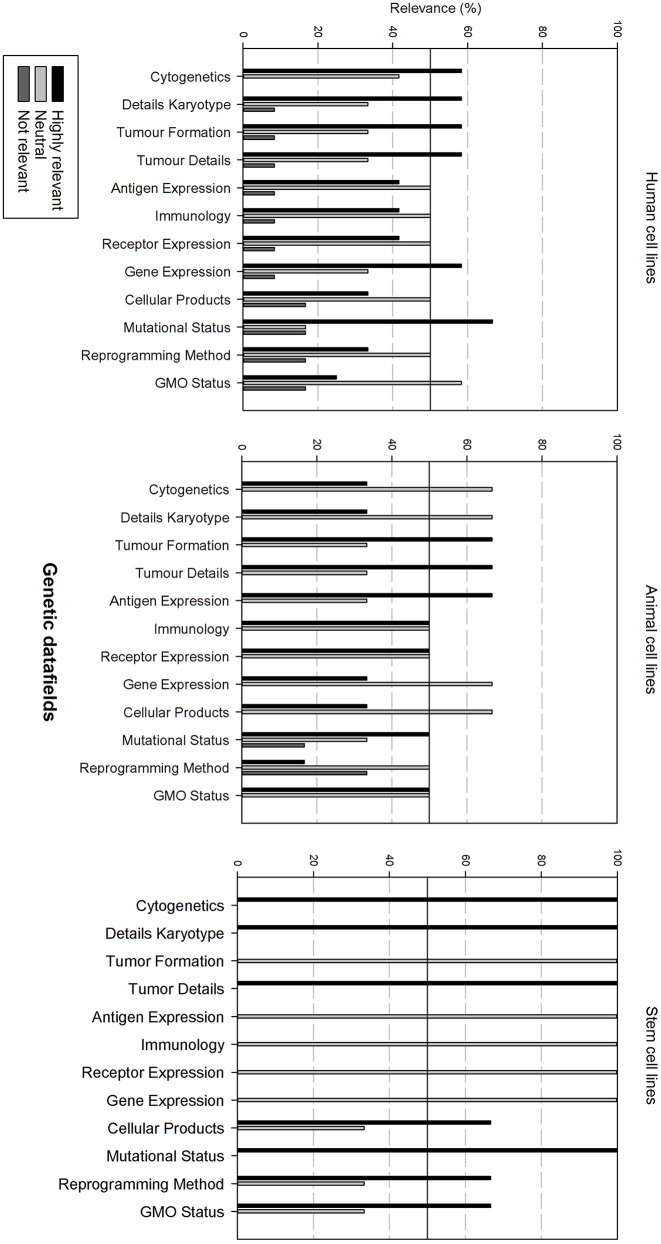
Overview of the relevance score of genetic data fields for each type of cell line. A cut-off line at 50% is visible, indicating which data fields are generally considered as highly relevant or as not relevant.

Figure 6 gives an overview of the genetic data fields. There is overall variation in fields that are considered as relevant between all cell line types. In general, for each cell line type, half of the data fields is considered as relevant, the other half as neutral.

**Figure 7:**
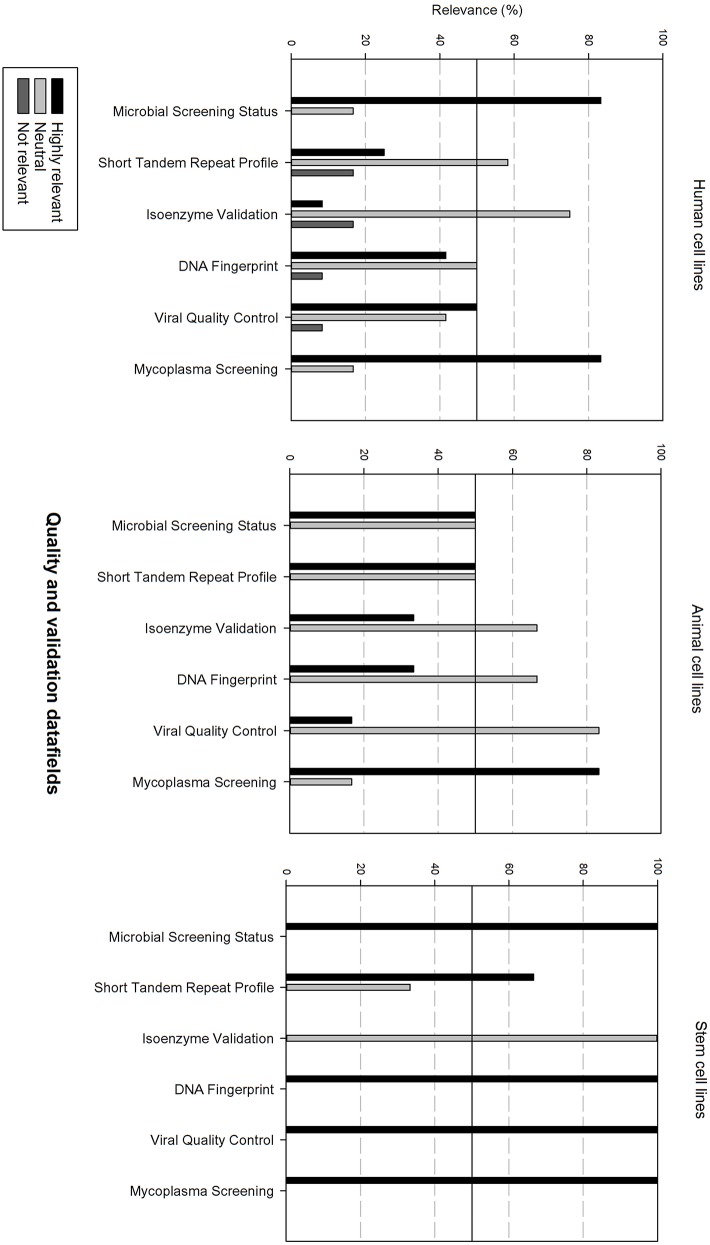
Overview of the relevance score of quality and validation data fields for each type of cell line. A cut-off line at 50% is visible, indicating which data fields are generally considered as highly relevant or as not relevant.

Figure 7 gives an overview of the quality and validation data fields. Microbial screening status and mycoplasma screening are considered as highly relevant for all cell types. Viral quality control is also considered as highly relevant for human cell lines and stem cell lines. STR profile is also rated as highly relevant for animal cell lines and stem cell lines. Additionally, DNA fingerprinting is also highly relevant for stem cell lines. The other data fields are considered as neutral.

**Figure 8:**
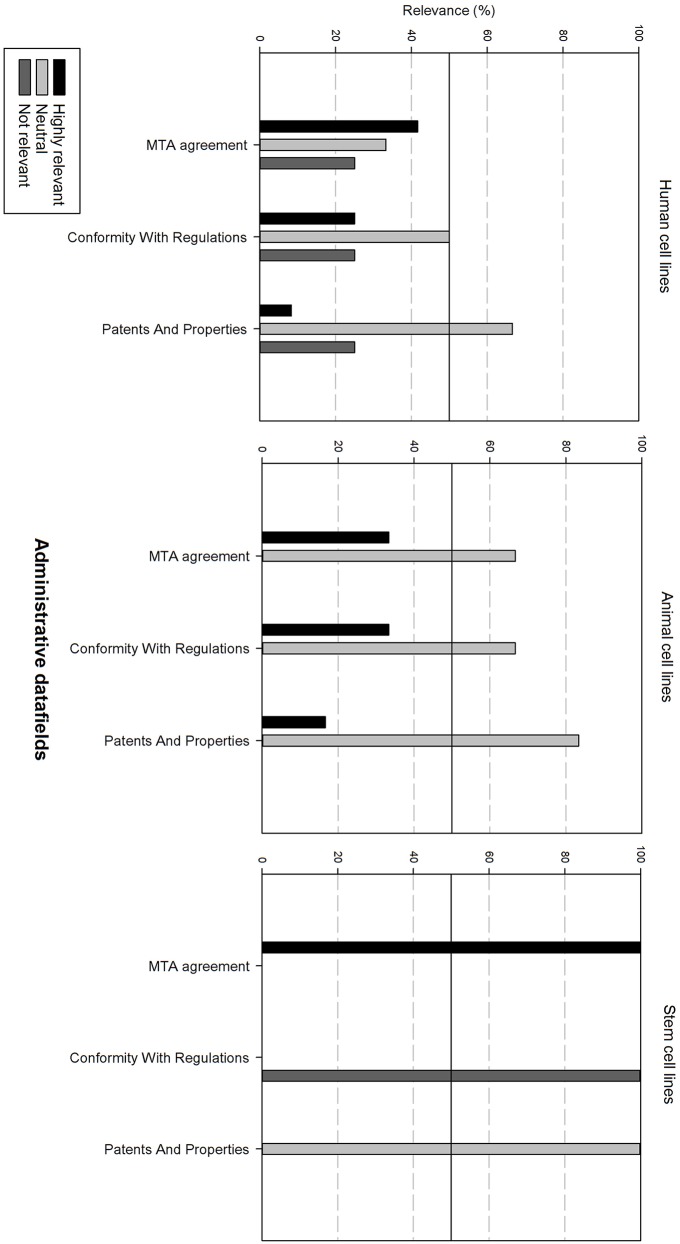
Overview of the relevance score of administrative data fields for each type of cell line. A cut-off line at 50% is visible, indicating which data fields are generally considered as highly relevant or as not relevant.

Figure 8 gives an overview of the administrative data fields. For human and animal cell lines, these are generally considered as neutral. For stem cell lines, the Material Transfer Agreement (MTA) is highly relevant. However, conformity with regulations is regarded as not relevant.

As analysis of the relevance of the data fields between human, animal, and stem cell lines showed that no parameters are deemed completely irrelevant in either type of cell line and a large overlap in relevance exists. Thus, we concluded to develop one dataset, useable for all three types of cell lines. All fields were retained and categorized into four levels. The basic and crucial data fields (level 1) consist of highly relevant fields, mandatory for all cell lines. The fields containing data related to certain procedures, quality processes or performed analysis can be found in level 2 and are considered as optional to fill in. Level 3 data is data pertaining to biobanking activities, such as operational, administrative and storage information. These data fields are completed by biobank staff members, and are also considered mandatory to fill in. Finally, all data that can be calculated or automatically filled in by the BIMS system has been classified as level 4 data.

From a GCDMP perspective, the data fields, regardless of their level, were further reviewed one by one,: units were separated from numbers and fields in which grouped data responses were expected were divided in multiple fields to capture one type of data per field. This led to an increase in amount of data fields in the dataset, though a better resulting data quality. Next, a clear and understandable “label” or “field name” was selected and a definition was added, to clarify the intended data response. Overall, this approach resulted in multiple changes in the dataset.

The “Organism” field has been relabeled to “Species” reducing ambiguity. In addition two extra fields -“Class” and “Order”—were added, which are automatically filled in when the species is selected into the “Species” field. Further, the fields “Tissue origin” and “Morphology” were evaluated and encompassed by the following fields: “Cell type,” “Cell line name” and “Anatomical location.” The field “Biosafety” was relabeled to “Biosafety level,” “Illness” to “Disease” and the fields “Age,” “Gender,” “Passage number” and “Ethnicity” were preserved. “Amount and cell concentration” were split into the fields “Amount (volume),” “Amount (volume) unit,” “Amount of cells,” “Amount of cells unit,” and two calculated fields “Cell concentration” and “Cell concentration unit.”

Multiple cell culture data fields were adapted for good data capture. “Adhesion” was relabeled as “Growth mode” as this field captures information regarding adherent or suspension culture and the label was deemed more appropriate. The field “doubling time” was split into a field capturing the number and the time unit. The fields “Culture atmosphere,” “Culture temperature” and “Antibiotic resistance” were kept as is and the field “Antibiotics” was changed to “Antibiotic addition” and “Medium renewal” was relabeled to “Medium renewal frequency” in order to avoid misinterpretation. The fields “Growth medium composition” and “growth medium additives” were subdivided in multiple fields labeled: “Basal culture medium,” “Serum (or alternative),” “% of Serum (or alternative),” “Growth medium additives,” “Growth factors” and “Remarks on culture medium.” “Sub cultivation ratio” was renamed to “Split ratio” and “subculture protocol” was renamed to “Cell dissociation agent or technique.” A similar approach was applied to the field of “Freezing medium composition.” The following fields were created to encompass all data: “Basal Freezing medium,” “Serum (or alternative) in freezing medium,” “Cryoprotectant,” “% of cryoprotectant” and “Freeze protocol.” “Freezing storage temperature” is renamed to “Storage temperature” and “Cryovial type” to “Storage container.” A date and time stamp “Freeze date and time” was also added to enhance the data value. The field “Thawing method” was split into “Basal thawing method,” “Serum (or alternative) in thawing medium,” “% of serum (or alternative) in thawing medium, “Thawing stabilizer,” “% of thawing stabilizer” and “Thawing temperature.”

To obtain clear data in the database, the fields related to quality control and genetic information were often split in multiple fields where the first field indicated if the analysis was performed and the second field with which technique/method, e.g., “Mycoplasma screening” became “Mycoplasma screening” and “Mycoplasma screening method.” This applied for “Antigen expression,” “DNA fingerprint” and “Viral quality control.” The field “Gene expression” was split into multiple fields, as mentioned above however as this field encompassed more complex information, 5 fields were created to capture this in a structured way. The fields “Cytogenetics/karyotype,” “GMO status” and “Tumor formation” were defined and by the use of fixed options there was no need to further separate the fields.

Before the cell line dataset was released for use on our campus, some additional fields were added to allow the practical implementation. The field “provider” is a field to identify who is bringing in the samples. Each collaborator of the biobank receives a unique number from the Bioresource center upon signing of the service level agreement. Additionally, the collaborators can use the fields “Biobank subcollection ID,” “study specific patient ID,” “Adremanumber,” “Reference ID,” “collection center” and “Visit number” to further define specific information regarding their collection. The fields “Status” and “date and time of registration” are filled in by the biobank personnel, as is the information regarding the location of the samples which are defined by the fields “Location path,” “Location,” “Row” and “Column.” Some open text fields are added to capture important additional information: “Remark of group,” “Sample remarks” and “Comment.”

A section of fields to encompass information regarding 2D and 3D culture on biomaterials was also included to be filled in optionally, as there is a large biomaterial and tissue engineering consortium present on our campus which uses multiple cell lines for their experiments.

To have an easily fillable, consistent and searchable database, the use of “fixed choice fields” was introduced. If ontologies and standard classifications/lists were available, the user-friendliness was reviewed. E.g., for designing a list of cell types a concise selection of different cell types was made out of the “Cell Line Ontology: CLO” (28, 31) as reference. These cell types will be combined with their anatomical location in an additional data field, based on the topology code of the “International Classification of Diseases for Oncology” (29) and for ease of use the high level of anatomic location was implemented (“Lip” instead of “External upper lip” etc.). The applied ontologies and standard lists that were considered, can be found in Table 3, under column “Standards/principles for data quality.”

**Table 3:** Essential cell line dataset.

**Data field label**	**Level**	**Data type**	**Principles for data quality**	**Definition**
Species	1	Fixed choice	Biological classification (taxonomy) for class, order and species	Species from which the animal cell line was derived
Class	4	Automatic completion	Biological classification (taxonomy) for class, order and species	
Order	4	Automatic completion	Biological classification (Taxonomy) for class, order and species	
Cell type	1	Fixed choice	Cell Line Ontology (CLO)	Cell line cell type.
Cell line name	2	Free text field	The International Cell Line Authentication Committee (ICLAC)	Name of the (commercial) cell line.
Tissue origin/anatomic location	1	Fixed choice		Anatomical location/origin of the sample.
Biosafety level	1	Whole number		Biological safety levels are ranked from one to four and are selected based on the agents or organisms on which the research or work is being conducted. Each level builds up on the previous level, adding constraints and barriers. The classification of your organism can be checked at https://www.biosafety.be/content/tools-belgian-classification-micro-organisms-based-their-biological-risks
Growth mode	1	Fixed choice		Growth mode of the cell culture.
Disease	1	String (restricted format)	Diseases for Oncology (ICD-O)	ICD10 code of the studied disease where for the sample was collected, https://icd.who.int/browse10/2016/en
Gender	1	Fixed choice		This indicates the gender of the participant/animal. “Unknown” means information about the gender was missing, “Other” stands for transgender/gender neutral participants.
Ethnicity	2	Fixed choice		A large group of people who have the same national, racial, or cultural origins, or the state of belonging to such a group.
Study specific patient ID	2	Free text field		The link to the patient (according to the patient identification log) (pseudonomized).
Adremanumber	2	String (restricted format)		Directly identifying patient identification code provided by UZ Gent.
Reference Id	2	Free text field		
Collection center	2	Fixed choice		This field contains the location where the sample was collected from the patient. It allows identification of multiple collection centers (e.g., hospitals or general practice centers).
Collection date and time	2	Date	ISO 8601	Date and time of collection.
Consent status	1	Fixed choice		The consent status of the participant regarding the sample.
Sample status on arrival	1	Fixed choice		Status of your sample at arrival in the Biobank facility.
Visit number	2	Whole number		This contains the visit number. E.g., 0 stands for the baseline visit.1 is the first visit after the baseline visit.
Type	1	Fixed choice		This describes the content type of the sample.
Passage number	1	Whole number		A record of the number of times the culture has been subcultured, i.e., harvested and reseeded into multiple ‘daughter’ cell culture flasks.
Amount (volume)	1	Whole number		
Amount (volume) unit	1	SI units	International system of units (SI)	
Amount of cells	1	Whole number		
Amount of cells unit	1	SI units	International system of units (SI)	
Cell concentration	4	Calculated field		
Cell concentration unit	4	SI units	International system of units (SI)	
Culture atmosphere	1	Fixed choice		The controlled atmosphere in which the cells are cultivated (CO_2_/O_2_ levels).
Culture temperature	1	Fixed choice		The controlled temperature at which cells are cultivated.
Basal culture medium	1	Fixed choice		The basic unsupplemented medium which promotes the growth of many types of cells.
Serum (alternative)	1	Fixed choice		Serum or alternative that contains a complex array of protein components, essential for cell culture.
% of serum (alternative)	1	Decimal number		Percentage of serum used in culture medium.
Growth medium additives	1	Fixed choice		Additional supplements to the basic culture medium that provide optimal growth conditions for the specific cell line.
Growth factors	1	Fixed choice		Additional growth factors to the basic culture medium that provide optimal growth or differentiation conditions for the specific cell line.
Antibiotic addition	1	Fixed choice		Antibiotics that are added to routine culture medium.
Antibiotic resistance	1	Fixed choice		Antibiotics for which the cell line is resistent.
Remarks culture medium	2	Free text field		Extra information concerning the culture medium.
Cell dissociation agent or technique	1	Fixed choice		Agent or technique used for dissociation of cells.
Split ratio	1	Fixed choice		The divisor of the dilution ratio of a cell culture at subculture, e.g., 1/5.
Doubling time	2	Time	ISO 8601	The period of time required for the cells to double in amount.
Doubling time unit	2	SI units	International system of units (SI)	
Seeding cell density	1	Whole number		Density/concentration at which the cells are seeded after passaging.
Seeding cell density unit	1	SI units	International system of units (SI)	
Medium renewal frequency	1	Fixed choice		Frequency of culture medium renewal.
Cell Line Stability	2	Fixed choice		Indication of cell line stability.
Basal Freezing Medium	1	Fixed choice		The basic unsupplemented medium which forms the essential part of the freezing solution.
Serum (alternative) in freezing medium	1	Fixed choice		Serum or alternative that contains a complex array of protein components, used to supplement the basic freezing medium.
% of serum (alternative) in freezing medium	1	Decimal number		Percentage of serum used in freezing medium.
Cryoprotectant	1	Fixed choice		A cryoprotectant is a substance used to protect biological tissue from freezing damage.
% of cryoprotectant	1	Decimal number		Percentage of cryoprotectant used in the freezing medium.
Freeze protocol	1	Fixed choice		Technique used for freezing the sample.
Conservation	1	Fixed choice		
Basal thawing medium	1	Fixed choice		The basic unsupplemented medium which forms the essential part of the thawing solution.
Serum (alternative) in thawing medium	1	Fixed choice		Serum or alternative that contains a complex array of protein components, used to supplement the basic thawing medium.
% of serum (alternative) in thawing medium	1	Decimal number		Percentage of serum used in thawing medium.
Thawing stabilizer	1	Fixed choice		Supplements added to the thawing medium to stabilize the cells during the thawing process.
% of thawing stabilizer	1	Decimal number		Percentage of thawing stabilizer in thawing medium.
Thawing temperature	1	Fixed choice		Temperature at which the samples are thawed.
Adapted to 3D culture	1	Y/N; Fixed choice		Has the cell line been adapted to 3D culture?
Feederlayer	1	Y/N; Fixed choice		Is a feeder layer needed for cell culture of the cell line?
Feederlayer determination	2	Free text field		Which feeder layer is needed for maintaining the cell culture of the cell line?
Biomaterial (basic composition)	2	Fixed choice		
Biomaterial modification	2	Fixed choice		
Biomaterial coating	2	Fixed choice		
Remark of group	2	Free text field		Extra remarks related to the cell line.
Sample remarks (QC)	2	Free text field		Remarks concerning the quality of the specific sample.
Comment	2	Free text field		General comment (cannot contain identifying data).
Storage temperature	1	Fixed choice		Temperature at which sample is stored.
Storage container	1	Fixed choice		Type of container in which the sample is store for long term storage.
Freeze date and time	1	Date	ISO 8601	Date and time of freezing of the sample.
Cytogenetics/karyotype	1	Fixed choice		Method used for karyotyping/ Cytogenetic procedures.
Antigen expression	1	Fixed choice		Is there antigen expression within the cell line sample?
Type of antigen expression	2	Fixed choice		What type of antigen expression can be observed?
Method of antigen expression	2	Fixed choice		Method used for determining the antigen expression profile.
Reprogramming method performed	1	Fixed choice		Was the cell line obtained through a reprogramming process?
Reprogramming method	2	Fixed choice		Which method was used for reprogramming the cell/line.
GMO status	1	Fixed choice		Is the cell line classified as a genetically modified organism?
Microbial screening status (microbial contamination)	1	Fixed choice		Was the sample screened for microbial contamination and what was the result of this screening?
DNA fingerprint	1	Fixed choice	The American National Standards Institute (ANSI), American Type Culture Collection (ATCC)	Has a DNA fingerprinting method been performed?
DNA fingerprint (method)	2	Fixed choice	The American National Standards Institute (ANSI), American Type Culture Collection (ATCC)	Which screening method was used for DNA fingerprinting?
Viral quality control	1	Fixed choice		Has the cell line been screened for viral contamination?
Viral quality control (method)	2	Fixed choice		Which method was used for the viral quality control?
Mycoplasma screening	1	Fixed choice		Has the cell line been screened for mycoplasma contamination?
Mycoplasma screening (method)	2	Fixed choice		Which screening method was used for the Mycoplasma detection?
Tumor formation	2	Fixed choice		Ability for tumor formation.
Gene expression level	2	Fixed choice		
Gene expression level (test)	2	Free text field		
Gene expression analysis method	2	Fixed choice		
Gene expression overexpression method	2	Fixed choice		
Gene expression inhibition method	2	Fixed choice		
Provider	1	String (restricted format)		Provider number given by Bioresource center Ghent (unique for the biobank)
Biobank subcollection ID	2	Free text field		Field that can be used to indicate specific subprojects in which the samples are collected
Status	3	Fixed choice		Operational status of sample (Bioresource center Ghent)
Date and time of registration	3	Date	ISO 8601	Date of registration in the biobank
Location path	3	Fixed choice		Location of the sample including subdivisions (freezer, shelf, rack…)
Location	1/3	Fixed choice		Box number
Row	1/3	Fixed choice		Row within the box in which the sample is located
Column	1/3	Fixed choice		Column within the box in which the sample is located
Cell line formation	2	Free text field		Description creation of the cell line.

The resulting cell line data set consists of a total of 101 data fields. The majority of these fields (58 out of 101 data fields) are level 1 fields, thus mandatory to complete by the researcher. There are 32 optional (level 2) data fields that allow to enhance the data quality, 7 level 3 fields that are filled in by the biobank staff and four automatic calculated fields (level 4). The dataset fields were configured in our BIMS system. Through an Excel template with the configured option lists, the information can easily be received from the researchers and put into the BIMS system.

## Discussion

Cell lines are essential in translational biomedical science. Misidentification, culture mix-ups, authentication and annotation issues often occur, hampering and delaying the reliability and reproducibility of preclinical tests, which are mandatory before the initiation of actual clinical trials. Accurate documentation of cell line data in a state-of-the-art database system is critical to ensure the credibility, reproducibility, and translation of data and results from cell culture-based experiments (17).

The need for international standards to close multiple gaps in this field is obvious. In order to resolve this issue, the first steps to harmonization are being initiated as new standards are arising [human cell line STR profiling (32) (ASN-0002), DNA barcoding for animal cell lines (13) (ASN-0003)]. Additionally, an effort was made to make cell line misidentification more conspicuous with the establishment of “The International Cell Line Authentication Committee (ICLAC).” They established controlled vocabularies and ontologies for already existing cell lines. However, a reduction in complications and redundancies in the literature concerning cell lines didn't seem attainable (15).

At our campus, multiple cell lines are kept in biobanks. The need for a uniform, campus-wide cell line dataset that tackles issues regarding misidentification, annotation and poor culture follow-up is high. We initiated this process by a large-scale literature and public database review of cell line datasets. There is an enormous lack of clear information in literature regarding cell line datasets and the fields these contain. A compiled extensive dataset was established as described in the Materials and Methods section. There is a massive difference in available information in the datasets pertaining to cell lines, as some vendors/repositories only list 8 data fields and others over 50 data fields regarding the same cell line. Further analysis through the redundancy strategy approach, revealed additionally a lack of standardization in terminology and definitions of the data field and the use of divergent labels for identical field information. It is clear that currently, different cell line repositories have established their own divergent sets of data fields without any verification or mutual agreement on which data should be recorded.

Through use of the redundancy strategy, a concise set of 54 data fields could be compiled for the survey dataset. As our aim was also to examine which data researchers are currently registering and which quality checks they are performing, we included these questions into the REDCap survey. The REDCap tool allows integration of all these parameters in a survey of reasonable length, which can be completed in a user-friendly way by the researchers. No comments about the setup of the survey or any remarks about difficulties completing the survey were received. Responses from human, animal, and stem cell line users were received, which allowed us to evaluate different expectations and needs regarding the datasets for human, animal and stem cell lines. Remarkable, over 75% of the cell line users do not authenticate their cell lines.

A general consensus could be observed regarding the high relevance of the basic cell line data fields, which was expected. These fields were also present in the datasets of the majority of all vendors/cell line depositors. The relevance of the clinical and demographic related data fields varies more, but is considered more as neutral. Cell culture information is considered as highly relevant or neutral. The pattern of relevance for human and animal cell lines is quite comparable. There is more distinction with stem cell lines, where certain parameters are considered as either very relevant or completely neutral. Some vendors/cell line depositors give only minimal information related to cell line culture parameters, or allow for the upload of culture protocols to be distributed upon request of the cell line.

Genetic data information is in general less prominent in datasets of vendors/cell line depositors, and is also mostly considered as neutral to relevant. Genetic information is prominent available when buying specific animal cell line clones, engineered for certain research purposes. It is clear however, that information related to the mutational status is necessary for stem cell lines. Quality and validation information is rarely available. It is assumed that some large vendors have quality and validation procedures in place, though no specific information can be given upfront, only upon request. One exception clearly standing out is the German collection of Microorganisms and cell cultures, hosted at the Leibniz Institute^8^. Clear information regarding the performed tests and results can be found online, e.g., Mycoplasma screening by PCR, DNA fingerprinting and type of performed PCR for revealing the STR profile, PCR analysis for several viral contaminants. This is crucial information when the cell lines are used in pre-clinical assays. Administrative information detailing the proposed use, warranties, limitations and restrictions of the material are considered as neutral. From a legal perspective, however, this is an exceptional important part of information and essential to keep track of, therefore these data fields will be kept in the developed dataset. Our analysis showed that the development of one dataset for the different types of cell lines would be applicable and usable for our local cell line users.

A list of data fields was compiled, based on the REDCap survey. Within this list, every single entry was subsequently evaluated based on score, relevance and multiplicity. In the survey, certain broad terms were used to describe the content of data field. These fields were split in more clearly definable sub-data fields according to GCDMP rules.

Two essential aspects to obtain good structured data are clear and unambiguous naming of the defined data fields and thoughtfully chosen definitions of the data fields.

The definitions implemented were based upon terminology used in three known biobanking data categorizing systems: SPREC, BRISQ, and MIABIS. SPREC and BRISQ are both proposed as a set of recommendations for reporting data elements of human biospecimens used in biomedical research with the difference that SPREC allows generating a code based on the pre-analytical processing of the samples (33). MIABIS, as its name suggests, is an attempt to unify sample data in a way that simplifies communication and exchange of samples (and sample information) in a clear and non-ambiguous way (6). The applicable definitions out of these standards were retained for implementation, although sometimes the formulation was simplified. Additionally, specific definitions that were not present in the standards were designed (e.g., especially for cell culture specific fields) based upon general accepted definitions out of histology and cell culture handbooks (34, 35).

The data quality was further enhanced through the implementation of standards and ontologies, and through the use of fixed fields, thus allowing limited options per field. Another measurement undertaken to maintain good data practice was the possibility to distinct missing data from empty “not filled in” data fields. For instance, within the selection list of the fixed choice data fields an option for missing data, e.g., not performed, unknown etc., was included. It is taken into account, however, that certain options might be to restraining to be able to input the data, thus, if this would occur, the biobank collaborators have the possibility to request additional options for a field. The request will be reviewed by a cell line advisory board, consisting of data managers and cell line experts. This is especially true for new and rapid expanding fields of research, for example the use of (bio)polymers in tissue engineering. Bearing this in mind, the complete data set will be implemented on campus with notification of the possibility to propose relevant additions to fixed choice fields. The complete set of defined options can be procured by contacting the Bioresource center manager.

The essential dataset consists of certain fields that are linked to each other, e.g., “Class,” “Order” and “Species.” To enhance the user-friendliness of the dataset, it was decided, through gathered experience weighed against literature-based study^9^ (36), to include the most commonly used organisms in research studies and not entire ontology lists. Additionally, when selecting the “Species” in the dataset, the related “Class” and “Order” are automatically completed in the database. This automatic completing of fields is also the case for the calculation of “cell concentration” and the unit based on “volume” and “amount of cells”.

For ethnicity (37), which is more important for human based cell lines, a compact classification was designed based on existing classifications^10^ (38) and the most common nationalities in Belgium (39–41). Statistics concerning citizens with a different ethnical background residing in Belgium can be found online, provided by the government. We finally designed a compact list of 29 different ethnicity options adapted to the Belgian population including persons who identify themselves with more than one social group. In case the Belgian population changes, extra options may be added. Other fixed choice lists were based on existing data fields from other data capturing systems.

Although useful as they are, and taking into account GCDMP guidelines, most of the withheld SPREC data fields needed to be subdivided to create clean and unambiguous data. For example, the SPREC field “Long term storage” which contained the temperatures at which a sample can be stored, the methods of storing the sample (Liquid Nitrogen (LN), Ultra low temperature (ULT) freezing, …), as well as the type of container used for storing the sample had to be split. It was subdivided into “Conservation,” encompassing the type of conservation (LN, ULT,…), “Storage temperature” en “Storage container.” In the same way the fields “Type of collection” (SPREC) and the non SPREC fields “Mode of transportation” and “Thawing procedure” were divided into numeric (temperature, time, …) fields and full text (protocol) fields. The use of these existing standards by researchers globally increases harmony and quality in biospecimen reporting in general.

Only minimal clinical and demographic information is present in our cell line dataset. We do, however, recognize the importance of this type of data relating to biobank samples but chose to collect these data in clinical registries, linked with the BIMS system. For clinical registries, REDCap, the tool that was used to perform the survey, can also be used. It allows capturing clinical and demographic information in a structured and easy way, by designing multiple forms that can be filled in at different time points. Additionally, sample data can be linked to the relevant clinical and demographic information present at that specific time point.

We chose to combine a system for sample data with one for clinical and demographic information, thus storing all the relevant data necessary for research in divergent but interconnected systems. This differs from other data capturing methods such as BRISQ where both sample and clinical/demographic information are encompassed within all three tiers (levels of importance to report) in one system. The main advantage of this type of data collection lies in the fact that all data concerning the sample is linked to the sample itself. The downside however, is that potentially large amounts of data are stored per sample. Additionally, many of the fields within the BRISQ tiers allow for free text data input, opening the possibility of clouding the data through less than optimal data management practices.

Furthermore, in Europe, addition of clinical and demographic data could make it possible to identify patients by specific information, such as birthdate, specific disease etc., which is not conform the General Data Protection Regulation guidelines. Thanks to the REDCap software, a clear distinction can be made between clinical and demographic data and the essential pre-analytical data concerning the sample itself in accordance with local legislation. This allows for researchers to use the REDCap tool as a Case Report Form and put an extra focus on sample specific data, which all too often is only an afterthought in the data capturing process. Altogether, a separation between clinical/demographic information and pre-analytical sample specific data will elevate sample-specific data quality and improve reproducibility.

The cell line dataset that was created, captures the most important information related to cell lines. The database allows distinction between clones of cell lines cultured in different settings, frozen and thawed with different procedures, products and methods and those that were kept on feeder layers, coatings or biomaterials. Additionally, the inclusion of genetic information and quality information makes the dataset extremely valuable.

Capturing this informations assures that cell line reactions observed in preclinical tests are in reality related to the performed test and not to “metadata,” meaning to possible contamination of the cell line, misidentification of the cell line or culture mix-ups. Clear cell line identification, in which genetic parameters of the cell lines are included, also lead to the correct use of the cell lines combined with certain specific test substances created for personalized medicine approaches or disease-specific solutions.

The dataset will be evaluated after one year of use. A customer satisfaction survey will be sent out to all our cell line users, who hopefully will be enthusiastic about the changes made. Based upon their feedback, additional changes could be made to the survey. As our biobank is part of the Biobanking and Biomolecular Resources Research Infrastructure of Belgium^11^, we will discuss spreading this dataset within our network to allow a broader use within Belgium as part of the ongoing harmonization strategies related to data management and quality.

## Data Availability

All datasets generated for this study are included in the manuscript and/or the supplementary files.

## Author Contributions

VT and LV devised the project, the main conceptual ideas, and proof outline. VT, LV, SV, SP, and EB worked out the details of the essential dataset, researching ontologies, known standards, and other literature. LV, SV, and SP worked out the actual technical implementation in the BIMS system upon agreement on the dataset in all its details. SB and CV supervised the project. VT wrote the main manuscript. All authors provided critical feedback and helped shape the research, analysis, and manuscript.

### Conflict of Interest Statement

The authors declare that the research was conducted in the absence of any commercial or financial relationships that could be construed as a potential conflict of interest.

## Footnotes

^1^Genohm, https://www.genohm.com/

^2^http://www.bbmri-eric.eu/

^3^http://www.isber.org

^4^https://www.labome.com/method/Cell-Lines-Companies.html

^5^https://www.uzgent.be/nl/home/Paginas/home.aspx

^6^https://eugdpr.org/

^7^https://www.iso.org/obp/ui#iso:std:iso:8601:-1:dis:ed-1:v1:en

^8^DSMZ: https://www.dsmz.de/home.html

^9^https://www.thermofisher.com/be/en/home/technical-resources/cell-lines.html

^10^https://www.ethnicity-facts-figures.service.gov.uk/ethnicity-in-the-uk/ethnic-groups-and-data-collected

^11^https://www.bbmri.be/
